# Simultaneous Modulation of Mesoporosity and Al Siting for Superior Performance Zeolite Catalyst in Ethylene Dehydroaromatization to Aromatics

**DOI:** 10.1002/anie.202508909

**Published:** 2025-07-29

**Authors:** Yanfeng Shen, Zhengxing Qin, Antoine Beuque, Eddy Dib, Shunsuke Asahina, Natsuko Asano, Izabel Cristina Medeiros Costa, Ruizhe Zhang, Lijuan Wang, Jiani Xu, Hongjuan Zhao, Jiujiang Wang, Ludovic Pinard, Svetlana Mintova

**Affiliations:** ^1^ State Key Laboratory of Heavy Oil Processing, College of Chemistry and Chemical Engineering China University of Petroleum (East China) No. 66, West Changjiang Road, Huangdao District Qingdao 266580 China; ^2^ Laboratoire Catalyse et Spectrochimie Normandie Univ, ENSICAEN, UNICAEN, CNRS 6 Bd Marchal Juin Caen 14000 France; ^3^ Advanced Material Analysis Co‐creation Research Center, Institute of Multidisciplinary Research for Advanced Materials Tohoku University Sendai Japan; ^4^ Application planning group JEOL Ltd. 3‐1‐2 Musashino Akishima Tokyo 196–8558 Japan; ^5^ Petrochemical Research Institute PetroChina Company Limited Beijing 100195 China

**Keywords:** Al siting, Crystal engineering, Ethylene dehydroaromatization to aromatics, Porosity, Zeolite

## Abstract

Porosity and Al siting are key levers for optimizing zeolite catalysts, yet they are often addressed independently due to the lack of effective integrated tuning strategies. Herein, we report an integrative methodology for preparing hierarchical zeolite (Z_F_) with interconnected mesostructures and tunable Al siting, achieved via regioselective dissolution of Al‐rich domains by presetting accessible and inaccessible zones within parent zeolite (Z_P_). Remarkably, ∼60% of the framework channels’ Al atoms were selectively removed without impairing the channel intersections’ Al atoms. Despite a 39% reduction in Brønsted acid sites (BAS), Z_F_ exhibits a 1.8‐fold higher turnover frequency (TOF) than Z_P_ during ethylene transformation at 973K, attributable to the introduction of mesoporosity. Notably, site‐specific ^31^P NMR analysis reveals that BAS located at channel intersections exhibits a TOF of 516 h^−1^, which is 13 times higher than that of sites within the channels (40 h^−1^). The hierarchical zeolite also demonstrates superior durability and enhanced aromatic selectivity compared to Z_P_. These results highlight the synergistic benefits of simultaneously tuning porosity and Al siting, offering a new paradigm for the rational design of high‐performance zeolite catalysts and providing deeper insights into the interplay between structure and function in zeolite‐based catalysis.

## Graphical abstract

A synergistic strategy enables the construction of hierarchical zeolites with selective removal of ∼60% of channel‐located Al, revealing that Brønsted acid sites (BAS) at channel intersections exhibit a TOF of 516 h^−1^, 13 times higher than those within the channels. This leads to a 1.8‐fold increase in ethylene conversion turnover frequency (TOF), enhanced aromatic selectivity, and superior stability at 973 K.

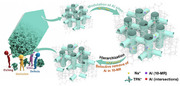

## Introduction

**Figure 1:**
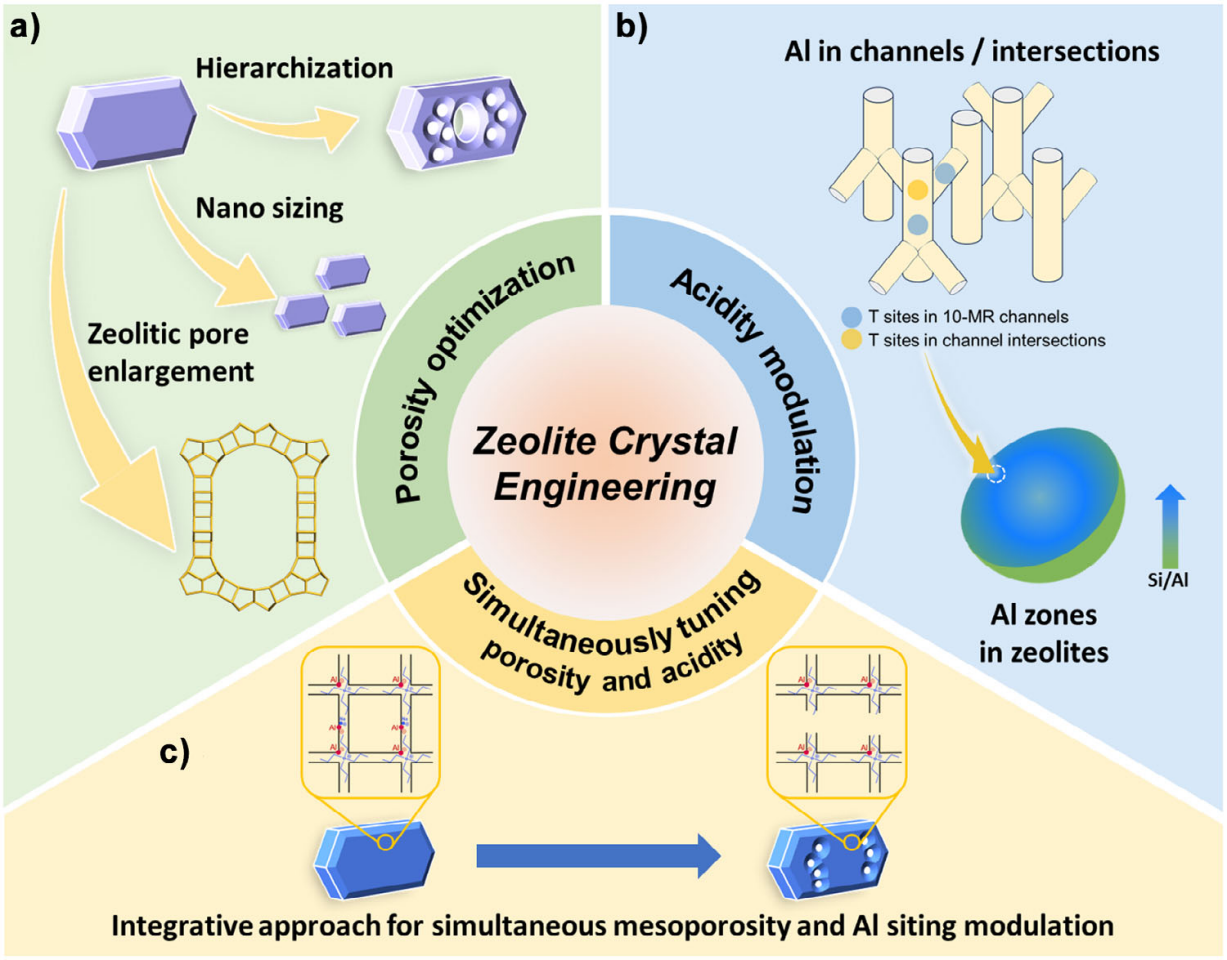
The prevailing methodologies for upgrading zeolite performance through crystal engineering: a) porosity optimization; b) Al siting modulation; and c) the integrated engineering of zeolite porosity and aluminum siting developed in this work.

Zeolites are microporous aluminosilicate materials exhibiting outstanding (hydro)thermal stability, tunable acidity, and unique shape selectivity. These integrated advantages make zeolites a compelling source of catalysts and sorbents in crucial industrial processes.^[^ ^1^, ^2^ ^]^ However, their performance is often constrained by mass transport limitations.^[^ ^3^, ^4^, ^5^ ^]^ The predominance of micropores leads to inherently low diffusion efficiency for bulky molecules, while the lack of precise control over active site distribution within the porous network further challenges the design of highly active and selective catalysts.^[^ ^6^ ^]^ To address these limitations, there are currently two *schools of thought* regarding the optimization of zeolite properties: i) diffusion features through porosity optimization and ii) acidity characteristics through the modulation of Al distribution (Figure 1a,b). The former enhances the molecular transport of bulky molecules and acid site accessibility, thereby improving catalytic activity.^[^ ^7^, ^8^, ^9^, ^10^ ^]^ while the latter fine‐tunes the spatial distribution and coordination of framework Al to improve catalytic selectivity.^[^ ^11^, ^12^, ^13^, ^14^ ^]^

Extensive research efforts have been devoted to optimizing zeolite pore architecture through three primary strategies, employing both bottom‐up and top‐down approaches: zeolite hierarchization,^[^ ^7^, ^15^, ^16^ ^]^ crystal nanosizing,^[^ ^17^, ^18^ ^]^ and zeolitic pore enlargement.^[^ ^19^, ^20^, ^21^ ^]^ (Figure 1a). In the domain of zeolites with expanded intrinsic porosity, the synthesis of ZMQ‐1, featuring 28‐membered ring pore openings, represents a recent and significant breakthrough.^[^ ^21^ ^]^ Conversely, in zeolite size minimization, structures have been refined to the scale of a single unit cell, pushing the limits of crystallite size reduction.^[^ ^18^, ^22^ ^]^ While these advancements mark the forefront of zeolite innovation, the development of hierarchical zeolites, particularly via post‐synthetic modifications, has gained considerable attention due to its practical feasibility and industrial compatibility.

Post‐synthetic modification enables the introduction of mesopores into existing zeolites through desilication, dealumination, or demetallation, with or without altering the overall framework composition.^[^ ^23^, ^24^ ^]^ These modifications frequently impact zeolite acidity, as selective extraction of framework Si or Al atoms,^[^ ^15^, ^25^ ^]^ or redistribution of aluminum species leads to varying degrees of acid site alteration.^[^ ^26^, ^27^ ^]^ The resultant changes range from negligible effects to significant enhancement or depletion of acidity, with some cases exhibiting synergistic effects due to the formation of extra‐framework aluminum species. Despite these advancements, the current level of control and understanding of acidity modification remains predominantly averaged, with acidity modifications often described in terms of acid site concentration, strength enhancement, or accessibility improvement.^[^ ^28^, ^29^, ^30^, ^31^ ^]^ The primary challenge in precisely modulating acid sites during the post‐synthetic engineering of mesoporosity arises from the limited control over zeolite framework hydrolysis. Although studies have shown that framework dissolution has a certain degree of preference, this level of tunability remains blurred.^[^ ^7^, ^26^, ^27^ ^]^ To date, there have been no reports demonstrating the precise tuning of aluminum siting during the post‐synthesis engineering of zeolite mesoporosity.

Achieving fine control over aluminum distribution and coordination necessitates alternative approaches.^[^ ^6^ ^]^ To name a few, the use of tetrapropylammonium (TPA^+^) cations preferentially locates framework Al at the intersections of the straight and sinusoidal channels of ZSM‐5.^[^ ^32^, ^33^ ^]^ The cooperative use of TPA^+^ and inorganic cations such as sodium (Na^+^) resulted in zeolite with Al sitting in both the intersections and in the channels of ZSM‐5.^[^ ^13^ ^]^ The use of bulky alcohol, such as pentaerythritol, in combination with Na^+^ resulted in zeolites with Al atoms located preferentially in ZSM‐5 channels.^[^ ^34^, ^35^ ^]^ The silicon source.^[^ ^36^, ^37^ ^]^ and the nature of the mineralizing agents also impact the placement of Al sites in distinct environments of ZSM‐5.^[^ ^38^ ^]^ Also, the inorganic cations (K^+^ and Na^+^) impact the framework Al arrangement in the CHA zeolite.^[^ ^39^ ^]^ In addition to one‐pot syntheses, several reports have been devoted to tuning Al siting through post‐synthesis dealumination.^[^ ^26^ ^]^ and re‐alumination.^[^ ^27^ ^]^ While numerous approaches were developed to tune framework Al siting at crystallographically non‐equivalent positions (Figure 1b), the spatial distribution of these site‐specific Al at the level of the crystallites is less addressed. However, it has been shown that the meso‐scale element zoning significantly influences the mass transport and catalytic properties of zeolite crystals.^[^ ^4^, ^40^ ^]^

Achieving a more comprehensive and precise understanding of the spatial distribution of Al at both micro‐ and mesoscopic levels marks a significant step forward in the rational design of next‐generation zeolite catalysts. Moreover, although both the engineering of zeolite porosity and the tuning of framework Al siting are crucial objectives,^[^ ^6^, ^8^ ^]^ a simultaneous modulation of these two properties through a single treatment is rarely achieved. The tuning of zeolite mesoporosity and Al siting at the same time not only enhances their catalytic properties through synergistic effects but also deepens the fundamental understanding of the intricate interplay between porosity and acidity. This study aims to develop an integrative approach that combines the engineering of mesoporosity and aluminum siting in zeolite catalysts (Figure 1c). To achieve this, several complementary strategies are employed: i) utilizing both organic and inorganic structure‐directing agents for the synthesis of the initial zeolite samples, ii) applying fluoride etching to achieve unbiased dissolution of partial zeolite crystals, iii) leveraging the steric hindrance effects of bulky molecules to modulate zeolite solubility, and iv) harnessing the mediating role of framework defects to guide the spatial distribution of mesoporosity. The catalytic performance of the outcomes of this integrative strategy was evaluated through ethylene transformation under severe conditions, highlighting their potential for enhancing zeolite catalytic activity and stability in demanding applications.

## Results and Discussion

The initial “parent” zeolite serves as a crucial foundation for subsequent structural modifications; it is this rationally designed synthesis that enables precise control over mesopore architecture and aluminum siting through the cooperative methodology. Herein, the synthesis of the parent ZSM‐5 zeolite (Z_P_) and the NH_4_F‐treated sample (Z_F_) followed a previously reported procedure.^[^ ^41^ ^]^ Our prior in‐depth investigation into its structural characteristics provides a robust basis for developing a synthesis strategy that integrates bottom‐up and top‐down approaches, facilitating precise structural tailoring.

In a typical synthesis, sodium hydroxide (NaOH) and tetrapropylammonium hydroxide (TPAOH) were dissolved in deionized water and stirred to ensure complete dispersion. Aluminum nitrate nonahydrate (Al(NO_3_)_3_·9H_2_O) was then added to the solution and stirred for 30 min. Subsequently, tetraethyl orthosilicate was introduced, and the mixture was stirred overnight to ensure complete hydrolysis of the silica source. The final gel composition was: 1.0 SiO_2_:0.02 Al_2_O_3_:0.25 NaOH:0.1 TPAOH:4.0 C_2_H_5_OH:100 H_2_O. This gel was transferred into a Teflon‐lined stainless‐steel autoclave and hydrothermally treated at 443 K for 24 h. The solid product was recovered by filtration, washed thoroughly with deionized water, and dried at 373 K overnight. For the NH_4_F post‐treatment, 4 g of the as‐synthesized ZSM‐5 (without prior ion exchange or calcination) was dispersed in 120 g of 30 wt% NH_4_F solution, stirred at 323 K for 4 h, and then filtered, washed, and dried at 373 K overnight. Both Z_P_ and Z_F_ samples were subsequently calcined at 823 K for 12 h to remove organic templates. The final protonated (H^+^) forms of the zeolites were obtained via ion exchange with (NH_4_)_2_SO_4_ solution, followed by a second calcination at 823 K for 4 h.

**Figure 2:**
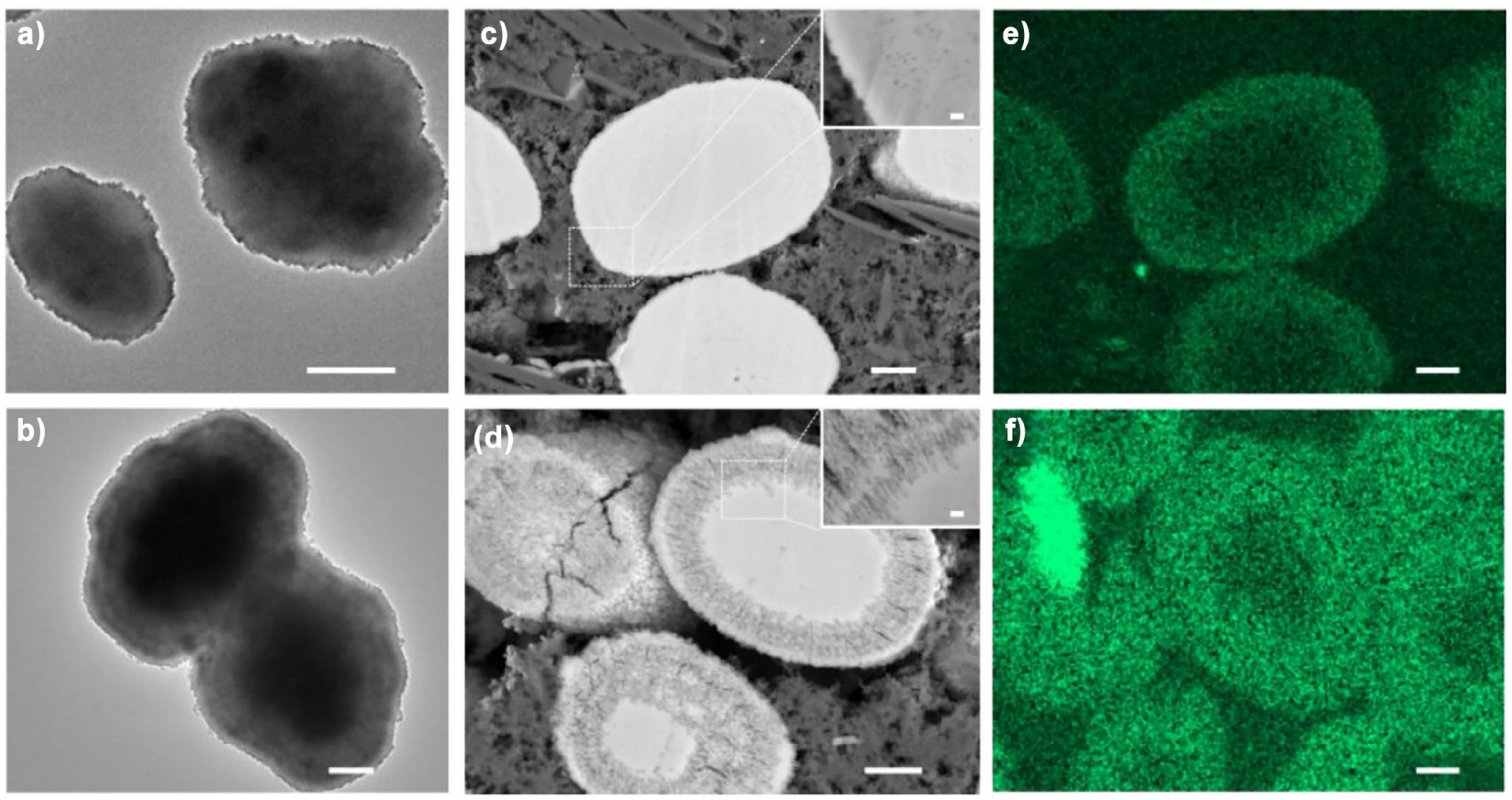
TEM images of Z_P_ a) and Z_F_ b). The cross‐section SEM images of the parent zeolite sample (Z_P_, c) and its NH_4_F treated counterpart (Z_F_, d), and the Al distribution over the cross‐section of samples Z_P_ e) and Z_F_ f). The scale bars for (a–f) in Figure 1 are 1 µm, and for the insets in (c) and (d) are 100 nm.

**Figure 3:**
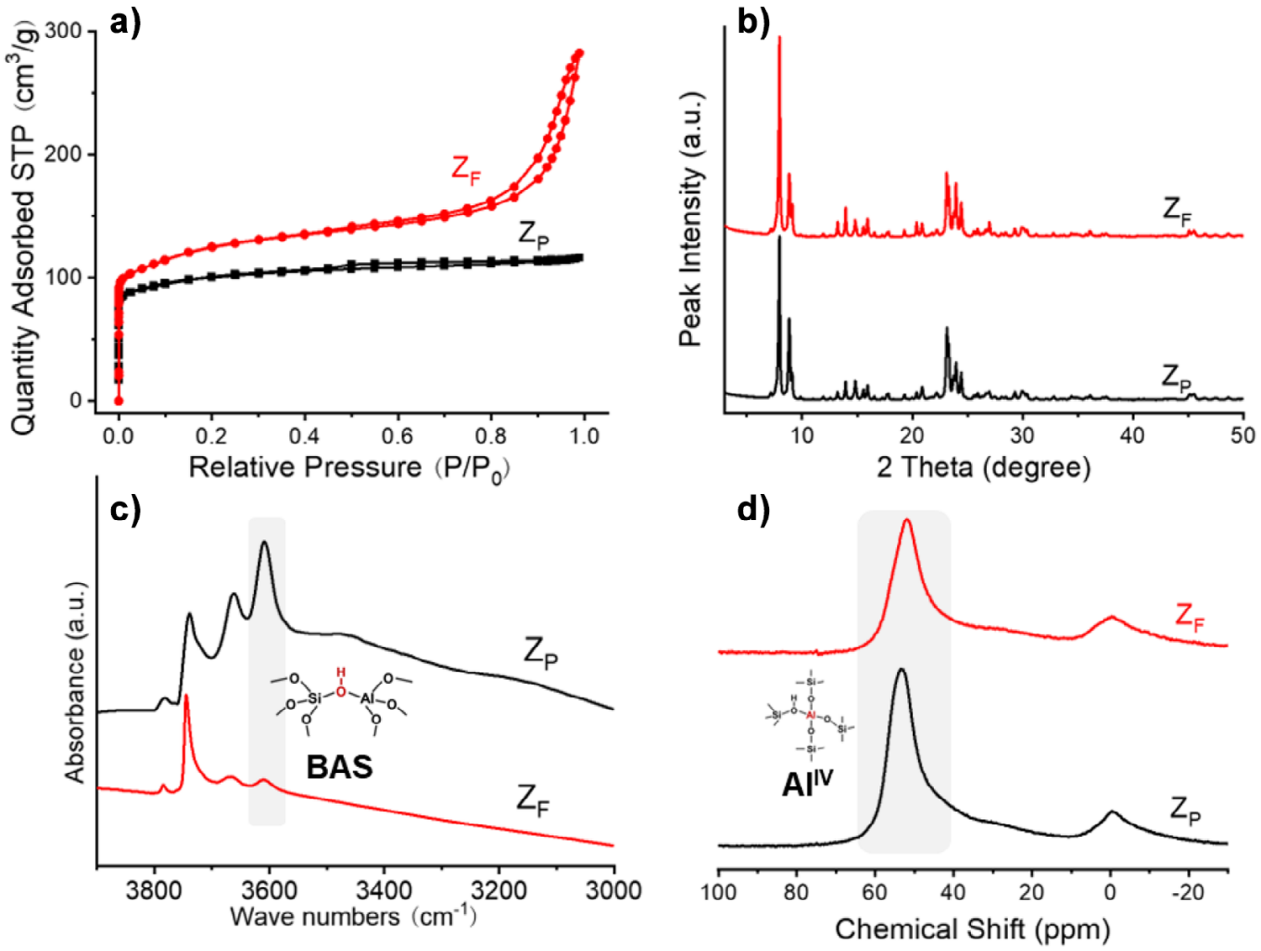
a) N_2_ adsorption‐desorption isotherms, b) XRD patterns, c) IR spectra, and d) ^27^Al NMR spectra of Z_P_ and Z_F_ zeolite catalysts.

The TPA^+^, located at channel intersections,^[^ ^42^ ^]^ imposed natural steric constraints that influenced the local accessibility of the etchant. NH_4_F was selected for its ability to extract both Si and Al in a chemically unbiased manner, without inducing the formation of extra‐framework aluminum species, thereby enabling well‐defined structural modification. Under the synergistic regulation of structural defects and the spatial constraints imposed by OSDA, this etching process induced regioselective crystal dissolution, generating radial dissolution tracks around a relatively intact core (Figure 2a–d). Consequently, Z_P_ was transformed into a highly porous framework with well‐developed mesopore connectivity (Figures S1–S3). Consistent with this observation, the textural properties underwent substantial changes. While Z_P_ exhibited a purely microporous structure (Figure 3a), Z_F_ evolved into a hierarchical architecture with pronounced mesoporosity (Figure 3a; Table 1), featuring a mesopore volume of 0.27 cm^3^ g^−1^. Nevertheless, despite the etching treatment, the crystallinity and microporosity of the sample were well preserved (Figure 3b; Table 1).

**Table 1:** The porosity data, bulk, and surface Si/Al ratios, and the acidity of Z_P_ and Z_F_ zeolite catalysts.

Sample	S_BET_ ^a^ ^)^ (m^2^ g^−1^)	V_micro_ ^b)^ (cm^3^ g^−1^)	V_total_ ^c)^ (cm^3^ g^−1^)	Si/Al^d)^	Si/Al^e)^	B_Py_ ^f)^ (µmol g^−1^)	L_Py_ ^f)^ (µmol g^−1^)
Z_P_	375	0.15	0.18	17.7	14.9	353	121
Z_F_	450	0.17	0.44	24.3	21.7	214	159

^a)^ The specific surface area calculated by BET method;

^b)^ the micropore volume calculated with t‐plot method;

^c)^ total pore volume obtained with the adsorption capacity of the N_2_ at the P/P_0_ = 0.98;

^d)^ the Si/Al measured by ICP;

^e)^ the Si/Al measured by XPS;

^f)^ the acidity analyzed by pyridine followed by FTIR.

Together with the generation of the open mesopores, the chemical etching results in a substantial decrease in the intensity of the hydroxyl bands associated with silanol nest‐type defects (3000 to 3500 cm^−1^; Figure 3c). In addition, the area of the peak attributed to the isolated silanols (3720 cm^−1^) decreased substantially in the Z_F_ catalyst. This observation provides clear evidence that defect regions are preferentially removed, facilitating mesopore formation^[^ ^7^, ^43^ ^]^ The generation of open mesopores is accompanied by an increase in the Si/Al ratio of the etched zeolite (Table 1). As a result, the amount of Brønsted acid sites (BAS, 3610 cm^−1^ in Figure 3c) decreased from 353 to 214 µmol g^−1^, while a slight increase in Lewis acidity was observed, rising from 121 to 159 µmol g^−1^. The higher framework Si/Al ratio in the NH_4_F‐treated zeolite crystal was further confirmed by the ^29^Si MAS NMR spectroscopy (Figure S4).

The apparent removal of framework Al and the corresponding increase in the bulk Si/Al ratio can be attributed to the preferential dissolution of Al‐rich zones.^[^ ^41^ ^]^ However, the observed regioselective dissolution patterns (Figures 2 and S1), combined with the spatially heterogeneous aluminum distribution (Figure 2e,f) and the marked alterations in the relative peak intensities of ^27^Al MAS NMR signals (Figure 3d), suggest collectively that the spatial organization of Al and Si exhibits a higher complexity than the traditional binary classification into Si‐rich and Al‐rich domains.

**Figure 4:**
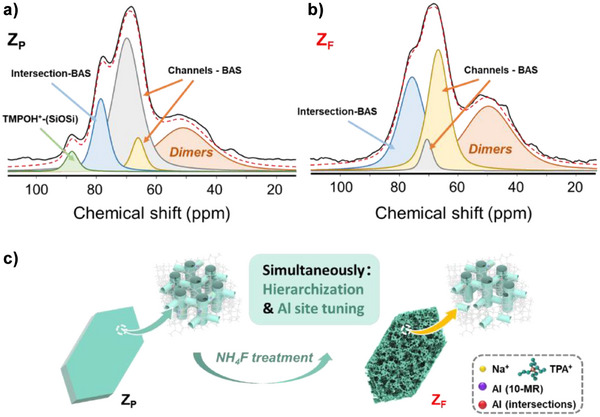
^31^P NMR spectra of samples Z_P_ a) and Z_F_ b) using TMPO; the peaks were attributed according to the work reported by Bornes et al.^[^ ^44^ ^]^ and the deconvolution results are summarized in Table 2. c) The schematic illustrates the simultaneous and selective removal of framework Al from ten‐membered ring channels and the formation of a hierarchical structure in zeolite crystals during NH_4_F treatment.

To gain deeper insight into this aluminum sites‐biased dissolution phenomenon, solid‐state NMR characterization was applied using TMPO as a probe molecule to localize the BAS in the zeolite catalysts, following the combined experimental and theoretical approach of Bornes et al.^[^ ^44^ ^]^ Based on the ^31^P NMR results (Figure 4), the interaction of TMPO with different sites in zeolites can be interpreted as follows: the TMPO dimers (broad peak ranging from 30 to 60 ppm), the molecules associated with Brønsted acid sites (BAS, at ca. 60 to 80 ppm), and the protonated TMPO species (ca. 88 ppm) at the intersection of the channels without interaction with BAS are identified (Table 2). The peaks in the range of 60–80 ppm can be further deconvoluted into three peaks, including the ones associated with the BAS in the zeolite channels (between 66 and 71 ppm) and those sitting in the intersections (75 to 79 ppm).

**Table 2:** BAS calculated by the deconvolution of the ^31^P NMR spectra.

Sample	Position (ppm)	Location‐interaction^a)^	Relative peak areas (%)^b)^	[BAS]^c)^ (µmol g^−1^)
Z_P_	51.1	Dimers	29.8	–
	66.1, 69.9	Channels‐BAS	52.2	275
	78.5	Intersections‐BAS	14.7	78
	88.2	TMPOH^+^‐(SiOSi)	3.3	–
	Total^c)^			353
Z_F_	49.8	Dimers	37.8	–
	66.8, 70.6	Channels‐BAS	33.5	115
	75.7	Intersections‐BAS	28.7	99
	Total^c)^			214

^a)^ Assignment of TMPO adsorbed according to Bornes et al.,^[^ ^44^ ^]^

^b)^ the relative peak areas derived from ^31^P NMR spectra deconvolution,

^c)^ BAS in channels and intersections of Z_P_ and Z_F_ determined by considering ^31^P NMR relative areas and FTIR overall concentration. The total amount of acidity is determined using pyridine as the probe molecule. The unit of acid measurement is µmol g^−1^ zeolite.

The ^31^P NMR quantification highlights a significant finding: despite challenges in distinguishing Al in sinusoidal and straight channels, the relative peak area ratio of “Channels‐BAS” to “Intersections‐BAS” decreases from 3.5 in Z_P_ to near‐equivalence (1.1) in Z_F_ (Table 2). Using pyridine probed Brønsted acid data (Table 1) as the reference for acid amount calculation, BAS quantified in Z_P_ channels and intersections are 275 and 78 µmol g^−1^, respectively (Table 2). In Z_F_, these values change to 115 and 99 µmol g^−1^. This quantification shows a reduction of 160 µmol g^−1^ in channel acid sites after NH_4_F etching, representing a removal of 58% of the sites. Conversely, BAS at intersections show an increase by 21 µmol g^−1^. These data suggest that the decreased acidity in Z_F_ is linked to preferential removal of framework Al atoms primarily located in zeolite channels. This quantitative assessment of the site‐specific removal of framework Al atoms, combined with the visualization of the regioselective dissolution pattern (Figures 2 and S1), allows us to map out a more comprehensive pattern of the internal structure of zeolites (Figure 4c). Three key structural features emerge. Firstly, consistent with prior report,^[^ ^41^ ^]^ framework Al atoms in TPA^+^ and Na^+^‐synthesized ZSM‐5 zeolites are found in both intersections and channels, confirmed by ^31^P NMR results (Figure 4). Secondly, we observe an inhomogeneous spatial distribution of Al, characterized by Al zoning (Figure 2e,f), in line with earlier studies.^[^ ^40^, ^41^ ^]^ Thirdly, our findings reveal a preferential enrichment of framework aluminum in dissolved zones, where Al is predominantly located within zeolite channels (Table 2). These zones, enriched with Na^+^ and alternating above and below undissolved TPA‐rich domains as indicated by the interweaving of mesopores and remaining crystalline domains (Figures 2b,d and S1b), demonstrate the preferential removal of Al‐rich domains accessible to the etchant. Consequently, the spatial distribution of TPA^+^ (Si‐rich) and Na^+^ (Al‐rich) in dissolved zones is mesoscopically heterogeneous. While the size and morphology of these alternating Al‐rich and Si‐rich domains warrant further investigation, the preferential removal of Al‐rich domains enriched with channel‐Al sites results in a hierarchical zeolite featuring radially oriented mesopores (Figure 3) and concentrated acid sites in intersections (Figure 4). This introduces a new paradigm for designing hierarchical zeolites with controlled Al distribution.

**Figure 5:**
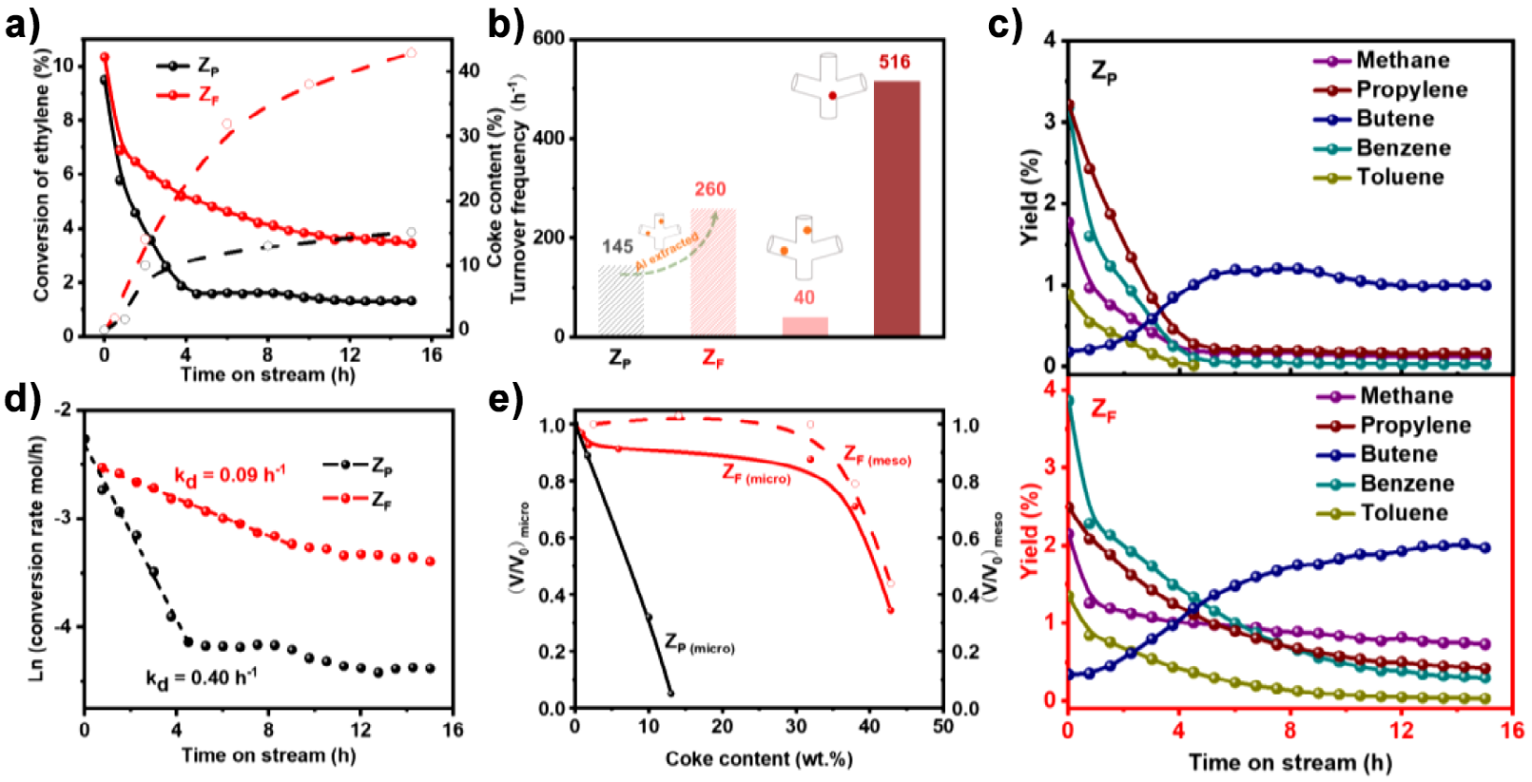
a) Ethylene conversion (solid balls) and coke content (empty circles) over Z_P_ (black) and Z_F_ (red) catalysts; b) TOF values of Z_P_, Z_F_, and the BAS sites in 10‐MR channels and channel intersections, respectively; c) molar yields of butenes (isobutene and but‐1‐ene), methane, propylene, benzene, and toluene as functions of time‐on‐stream at 973K, under atmospheric pressure, with diluted ethylene feed ($P_{C_{2}H_{4}}$ = 0.005 MPa) over Z_P_ (black) and Z_F_ (red) catalysts; d) deactivation rate of the Z_P_ and Z_F_ catalysts; e) evolution of residual micro‐ and mesopore volumes as a function of coke content. Reaction conditions: 973 K, atmospheric pressure; feed gas: ethylene/N_2_ mixture (ethylene partial pressure: 0.005 MPa); ethylene WHSV: 14 h^−1^; reaction duration: up to 16 h.

The catalytic consequences of hierarchical porosity and framework Al siting are examined using ethylene dehydroaromatization (EDA), an efficient model reaction for investigating the impact of zeolite acidity on the catalytic behavior.^[^ ^45^ ^]^ EDA over H‐ZSM‐5 features an induction period, attributed to the formation of an active hydrocarbon pool that facilitates the conversion of ethylene into aromatics. Ethylene transformation can proceed either via a sequence of pairing reactions involving confined and alkylated aromatic rings or through dimerization on BAS involving unstable primary carbenium ions (Figure S5). Under operating conditions (973K, atmospheric pressure, ethylene/N_2_ mixture with 0.005 MPa ethylene partial pressure as the feed gas, ethylene weight hourly space velocity (WHSV) of 14 h^−1^, and reaction durations ≤16 h), a blank test performed without a catalyst, relying solely on thermal conversion, resulted in less than 1% conversion of C_2_H_4_ to butenes (isobutene and but1ene), with a very small fraction undergoing further cracking to propylene and methane. The two catalysts achieve a comparable initial ethylene conversion of approximately 10% (Figure 5a). This low conversion can be attributed to the severe operating conditions employed. The high reaction temperature and low olefin partial pressure limit the oligomerization reaction.^[^ ^46^, ^47^, ^48^, ^49^ ^]^ For instance, over ZSM‐5 (Si/Al = 103) at 748K with a diluted ethylene feed (0.013 MPa), the conversion is only 2%,^[^ ^50^ ^]^ whereas at 623K with a C_2_H_4_ partial pressure of 0.1 MPa, over 83% conversion was reported.^[^ ^51^ ^]^

Despite a 39% lower concentration of BAS, the catalytic activity of Z_F_ is comparable to that of Z_P_, showing 1.8 times higher turnover frequency (TOF) per protonic site for Z_F_ (260 h^−1^ versus 145 h^−1^; Figure 5b). This enhanced catalytic performance is attributed to the generation of radial mesoporosity induced by the NH_4_F etching treatment (Figure 2). The aluminum distribution within the zeolite crystals, determined from the ^31^P NMR spectra, enabled the calculation of the TOF for BAS located in both the channels and the channel intersections. It turns out that BAS located at channel intersections exhibit a TOF of 516 h^−1^, which is 13 times higher than that of sites within the channels (40 h^−1^). This observation contrasts with previous studies on the role of acid site positioning in ZSM‐5. Protonic acid sites in the straight and sinusoidal channels are commonly associated with superior catalytic performance in reactions requiring molecular diffusion and shape selectivity,^[^ ^52^ ^]^ such as methanol‐to‐olefins (MTO) conversion.^[^ ^53^ ^]^ and butylene catalytic cracking.^[^ ^54^ ^]^ However, the significantly higher activity of BAS at the channel intersections observed in this study highlights their critical role under the severe reaction conditions investigated, characterized by high temperature and low partial pressure of ethylene, which differ markedly from the conditions employed in the cited studies. This underscores the importance of acid site location in determining catalytic efficiency under varying operational environments.^[^ ^11^ ^]^

Ethylene transformation at 973K produces aromatics (benzene and toluene), olefins (propylene and butenes), and methane (Figure 5c). The Z_F_ catalyst obtained by NH_4_F etching enhances the selectivity toward aromatics (Figure S6a). Additionally, the initial molar ratio of propylene to methane is close to 1 (e.g., 0.9), suggesting that ethylene primarily dimerizes into butene, which subsequently cracks into propylene and methane. In contrast, on the parent zeolite, the ratio is closer to 1.8, indicating that propylene is likely formed through a different reaction pathway, such as the alkene cycle observed in MTO processes. A similar dependence on Al siting has been observed in MTO catalysis. Liang et al. reported that Al located at channel intersections favors the aromatic‐based cycle, enhancing selectivity toward ethene and aromatics.^[^ ^36^ ^]^ Conversely, Al positioned in the sinusoidal or straight channels promotes the alkene‐based cycle, resulting in increased formation of propene and higher olefins. Wang et al.^[^ ^53^ ^]^ demonstrated through density functional theory calculations and molecular dynamics simulations that an increased number of acid sites in the sinusoidal and straight channels favors the alkene cycle, enhancing selectivity toward propylene. Therefore, the removal of acid sites located in the channels of the Z_F_ catalyst could explain the observed change in the initial selectivity.

Z_P_ catalyst undergoes complete deactivation after 4 h of reaction (Figure 5a), accompanied by a shift in selectivity from aromatics to propylene and butenes (Figure S6b). Upon complete deactivation, only the thermal dimerization of ethylene is observed. In contrast, despite a similar initial coking rate (Figure 5a), the Z_F_ catalyst exhibits a deactivation rate that is four times slower (Figure 5d) and retains the ability to catalyze ethylene aromatization even after 16 h of reaction (Figure 5c). Analysis of the coke composition reveals distinct differences between the two catalysts. On the hierarchical Z_F_ catalyst, soluble coke predominantly consists of polyalkylnaphthalene species even after 10 h of reaction (Figure S7). In contrast, on the parent Z_P_ catalyst, pyrene formation is detected as early as 4 h into the reaction. Moreover, most of the coke molecules are insoluble in dichloromethane, suggesting that on Z_P_, the coke is mainly composed of macromolecular carbonaceous species. Beuque et al.^[^ ^46^ ^]^ demonstrated that coke formed in micrometric zeolite HZSM‐5 with a Si/Al ratio of 40 evolves rapidly into heavy coke molecules. These heavy molecules, detected by LDI TOF, appear early during the process, with molar masses of 474, 556, and 684 m/z, corresponding to the compounds with chemical formulas of C_37_H_30_, C_43_H_40_, and C_53_H_48_, respectively.

Given that the number of BAS at the channel intersections is nearly identical in both samples (Table 2), the enhanced stability of Z_F_ is likely attributable to the introduction of radial mesopore connectivity and/or the reduced concentration of BAS in the channels. Acid sites in channel intersections, while providing larger spatial environments conducive to the formation of bulky intermediates, are also associated with undesirable reactions such as aromatization and hydrogen transfer. These reactions promote coke formation and accelerate catalyst deactivation.^[^ ^53^ ^]^

Despite its high resistance to deactivation, coke continues to accumulate on the Z_F_ catalyst (Figures 5a and S8), reaching a substantial content without significantly impacting the microporosity (Figures 5e and S9). This additional coke is not attributed to a thermal effect, as the coke content reaches a plateau at 12 wt% in the case of Z_P_ (Figures 5a and S10). A significant reduction in both micro‐ and mesoporosity is observed only at high coke contents exceeding 30 wt%. This coke, predominantly composed of alkylnaphthalene species, primarily acts as a spectator. In contrast, pyrenic compounds formed on both catalysts deactivate BAS at the intersections, contributing to catalytic deactivation. Numerous studies have demonstrated that coke accumulation within the mesopores of hierarchical zeolites obtained through alkaline treatment does not significantly affect the catalytic activity or stability.^[^ ^55^, ^56^, ^57^ ^]^ This behavior is attributed to the retention of coke by the abundant silanol groups generated during the alkaline treatment. In contrast, for the Z_F_ catalyst, the NH_4_F etching process does not generate such coke‐trapping sites (Figure 3c), indicating an alternative mechanism for coke retention. This retention is likely related to the high boiling point of the coke formed. This hypothesis is supported by a study on ethylene dehydrogenation to aromatic at 973 K over a micron‐sized zeolite.^[^ ^46^ ^]^ demonstrating that heavy coke forms on the external surface of the catalyst. This heavy coke, produced through condensation reactions, comprises more than 100 carbon atoms and has a molar mass exceeding 1300 g·mol^−1^, contributing to its high boiling point.

## Conclusion

In conclusion, a multi‐faceted strategy has been developed for the controlled construction of hierarchical ZSM‐5 zeolites with tunable Al distribution and mesoporosity. This was achieved through the combined effects of organic and inorganic structure‐directing agents, unbiased fluoride etching, steric regulation by occluded organics, and defect‐mediated mesopore formation. This approach enables the formation of predefined accessible and inaccessible domains, which guide the regioselective post‐synthetic dissolution of Al‐rich, Na^+^‐dominated regions while preserving TPA^+^‐protected Si‐rich domains. As a result, a spatially heterogeneous Al distribution emerges at the mesoscale, featuring alternating Al‐rich and Si‐rich nanosized domains within individual zeolite crystals. To the best of our knowledge, such a mesoscale structural organization has not been previously reported.

This spatial modulation directly leads to the formation of a hierarchical ZSM‐5 catalyst with a mesopore volume of 0.27 cm^3^ g^−1^ and an interconnected mesoporous network. Furthermore, the preferential removal of framework Al from the dissolved zones, particularly within the ten‐membered ring channels, results in the selective elimination of ∼60% of the channel‐located Al, while preserving Al atoms at the channel intersections. The simultaneous modulation of mesoporosity and Al siting results in a more active and durable catalyst, exhibiting enhanced aromatic selectivity in the EDA reaction at 973 K. Quantitative analysis of turnover frequencies associated with BAS in distinct structural environments reveals that those located at channel intersections exhibit a TOF 13 times higher than those within the channels. This pronounced difference underscores the dominant catalytic role of intersection‐confined acid sites under severe reaction conditions.

The combined strategy of selective framework Al removal and mesoporosity generation presents a broadly applicable approach for designing advanced zeolite catalysts. Beyond ZSM‐5 zeolite, this methodology could be extended to other frameworks such as BEA and MOR, where Al siting plays a decisive role in catalytic behavior and stability. In particular, the ability to tailor acid site location and accessibility opens avenues for optimizing reactions such as MTO and methanol‐to‐aromatics, by enhancing product selectivity and resistance to deactivation. The integration of mesostructuring and Al siting control may thus serve as a general pathway for improving both the performance and regenerability of zeolite‐based catalysts across a wide range of industrially relevant processes. Taken together, this work provides fundamental insights into the co‐engineering of spatially resolved acid site distribution and hierarchical porosity within a single zeolite framework, enabling enhanced catalytic performance under demanding conditions and deepening our understanding of structure–function relationships in zeolite catalysis.

## Supporting Information

Detailed experimental precedures were described in the Supporting Information.

SEM, TEM and SEM‐EDX mapping images, ^27^Al MAS NMR spectra, N_2_ (77K) sorption‐desorption resultsand thermal analysis curves can be found in the Supporting Information.

## Conflict of Interests

The authors declare no conflict of interest.

## Supporting information



Supporting Information

## Data Availability

The data that support the findings of this study are available from the corresponding author upon reasonable request.
