# Supplementary material for: Simultaneous Modulation of Mesoporosity and Al Siting for Superior Performance Zeolite Catalyst in Ethylene Dehydroaromatization to Aromatics
Source: Angew Chem Int Ed Engl. 2025 Jul 29;64(37):e202508909. doi: 10.1002/anie.202508909 (PMC12416449; doi:10.1002/anie.202508909)
Supplement: Supplementary file 1 — Supporting Information [file ANIE-64-e202508909-s001.docx]

**Supporting information**

Simultaneous modulation of mesoporosity and Al sitting for superior performance zeolite catalyst in ethylene dehydroaromatization to aromatics

Yanfeng Shen,^‡a^ Zhengxing Qin, ^‡^ *^a^ Antoine Beuque,^b^ Eddy Dib,^b^ Shunsuke Asahina,^c,d^ Natsuko Asano,^d^ Izabel Cristina Medeiros Costa,^b^ Ruizhe Zhang,^b^ Lijuan Wang,^a^ Jiani Xu,^a^ Hongjuan Zhao^e^, Jiujiang Wang^e^, Ludovic Pinard,^b^ Svetlana Mintova*^b^

**Characterization**

The crystallinity of the zeolite catalysts was measured on a Bruker D8 Advance equipped with Cu Kα radiation. The N_2_ (77 K) physisorption-desorption experiment was carried out with an Autosorb iQ3 (Qutantachrome). The specific surface area and micropore volume were calculated with BET (Brunauer-Emmett-Teller) and t-plot method based on the obtained isotherms, respectively. The mesopore volume was calculated by the total pore volume (V_total_) and micropore volume (V_micro_), where the V_total_ was obtained according to the quantity of N_2_ adsorption at the relative pressure of P/P_0_ = 0.98.

The morphology of the zeolite crystals was characterized by a low-voltage-high-resolution field emission scanning electron microscope (LVHR-FESEM, JSM-7900F, JEOL). The zeolite crystal cross-section was obtained using an ion-beam polisher (IB-19510CP, JEOL). The elemental distribution over the zeolite cross-sections was measured by an Oxford EDS detector (EDS 170 mm x 2). The procedure for sample treatment and data collection follows the method developed by Asahina et al. ^1^. The TEM images were obtained on a transmission electron microscope (TEM, JEM-2100, JEOL) with an accelerated voltage of 200 kV.

The Fourier Transform Infrared Spectrometer (FTIR) characterization was carried out on a Nicolet Magna 550-FT-IR spectrometer with an optical resolution of 2 cm^-1^. Prior to the experiment, the calcined zeolite samples were ion-exchanged with (NH_4_)_2_SO_4_ solution and calcined at 823 K to obtain the H^+^-form of the zeolite catalyst. The prepared H^+^-form zeolite catalysts were pressed into a self-supporting disc and heated at 823 K in a vacuum (10^-6^ Torr) for 5 h. The pyridine was used as a probe molecular for testing the acidity of the zeolite catalysts. The number of the Brønsted and Lewis sites was calculated based on the peak integration at 1545 cm^-1^ and 1454 cm^-1^, respectively of the FTIR spectra collected at the pyridine desorption temperature of 423 K.

All the magic angle spinning (MAS) nuclear magnetic resonance (NMR) spectra were measured on a Bruker Avance III HD 500 MHz spectrometer using a 4 mm probehead. The MAS frequencies were set at 12 kHz for ^29^Si NMR spectra and 14 kHz for ^27^Al and ^31^P NMR spectra, respectively. Single pulse experiments were used to acquire all spectra. π/3 (36 kHz), π /6 (67 kHz) for, and π/2 (33 kHz) pulses were used for ^29^Si ^27^Al ^31^P, (radiofrequencies between brackets). The numbers of scans were 1280, 4096, and 2048 for ^31^P, ^27^Al and ^29^Si NMR measurements, respectively. The recycle delays were 50s, 1s and 20s for ^31^P, ^27^Al and ^29^Si NMR measurements, respectively. The spectra were calibrated with respect to phosphoric acid (0.0 ppm) for ^31^P, aluminum nitrate (0.0 ppm) for ^27^Al, and tetramethylsilane (0.0 ppm) for ^29^Si NMR measurments.

The ^31^P NMR spectra of trimethylphosphine oxide (TMPO) probe molecules were acquired after adsorbing the molecules following the procedure described earlier^2, 3^. Typically, the pretreatment of the catalysts was performed in a glove box under Ar atmosphere to prevent any reaction between the TMPO and the air humidity or any evaporation. The zeolite catalysts heated at 673K under vacuum (4.0×10^-5^ Torr) were added in a solution of TMPO dissolved in dichloromethane (CH_2_Cl_2_). Similar P/Al molar ratio was used for both samples, i.e. 1.01 for Z_P_ and 1.16 for Z_F_. The mixture was then evaporated at room temperature under vacuum for 2 h and the treated zeolites were transferred into zirconia rotors.

**Ethylene aromatization over Z_P_ and Z_F_ zeolite catalysts**

The ethylene dehydrogenation to aromatics (EDA) reaction was carried out following the conditions described earlier^4^. The ethylene was diluted in a N_2_ flow at a 0.005 MPa partial pressure. The reaction was carried out at 973 K under ambient pressure. The weight hourly space velocity (WHSV) of the ethylene injected into the fixed-bed continuous flow quartz reactor was 14 h^−1^ (g of injected C_2_H_4_ per hour and per g of catalyst). In order to have a deep sight on the catalysis process, a series of reaction times (ranging from 0 - 16 h) were selected. The intermediate samples were investigated, including the porosity, the acidity and coke species.

**Characterization of intermediates and spent zeolite catalysts**

The porosity and acidity of the intermediates and spent zeolite catalysts were measured with N_2_ physisorption and pyridine-adsorbed FTIR. The only difference of the analysis between the fresh and used catalysts was the pretreatment conditions of the samples because of the coke species present in the used catalysts. Specifically, for the N_2_ adsorption and desorption analysis, the degassing of the samples was performed at 393 K for 12 h. And the pretreatment of the used catalysts subjected to FTIR measurments was carried out at 373 K under vacuum.

The coke content was determined using a thermogravimetric analysis (TGA) on a SDT Q600 TA Instrument. Prior to analysis, the samples were kept at room temperature under a dry air flow (100 mL/min) for 30 minutes. The temperature was then ramped to 1173 K at a rate of 10 K/min.

The coke species in the used zeolite catalysts were extracted following the method reported by Magnoux et al.^5^. 0.3 g used zeolite catalyst was dispersed in 3 ml of hydrogen fluoride (HF, 51%) and kept under stirring at room temperature for 30 min. The boric acid ([H_3_BO_3_] = 40 g/L, 18 ml) and sodium bicarbonate ([NaHCO_3_] = 20 g/L, 18 ml) solutions were sequentially added to the mixture to dilute and neutralize the solution. Afterwards, the hydrocarbon species in zeolite crystals were extracted by adding the dichloromethane (CH_2_Cl_2_) and identified by a GC-MS (Thermo Electron DSQ) equipped with a DB5 ms column.


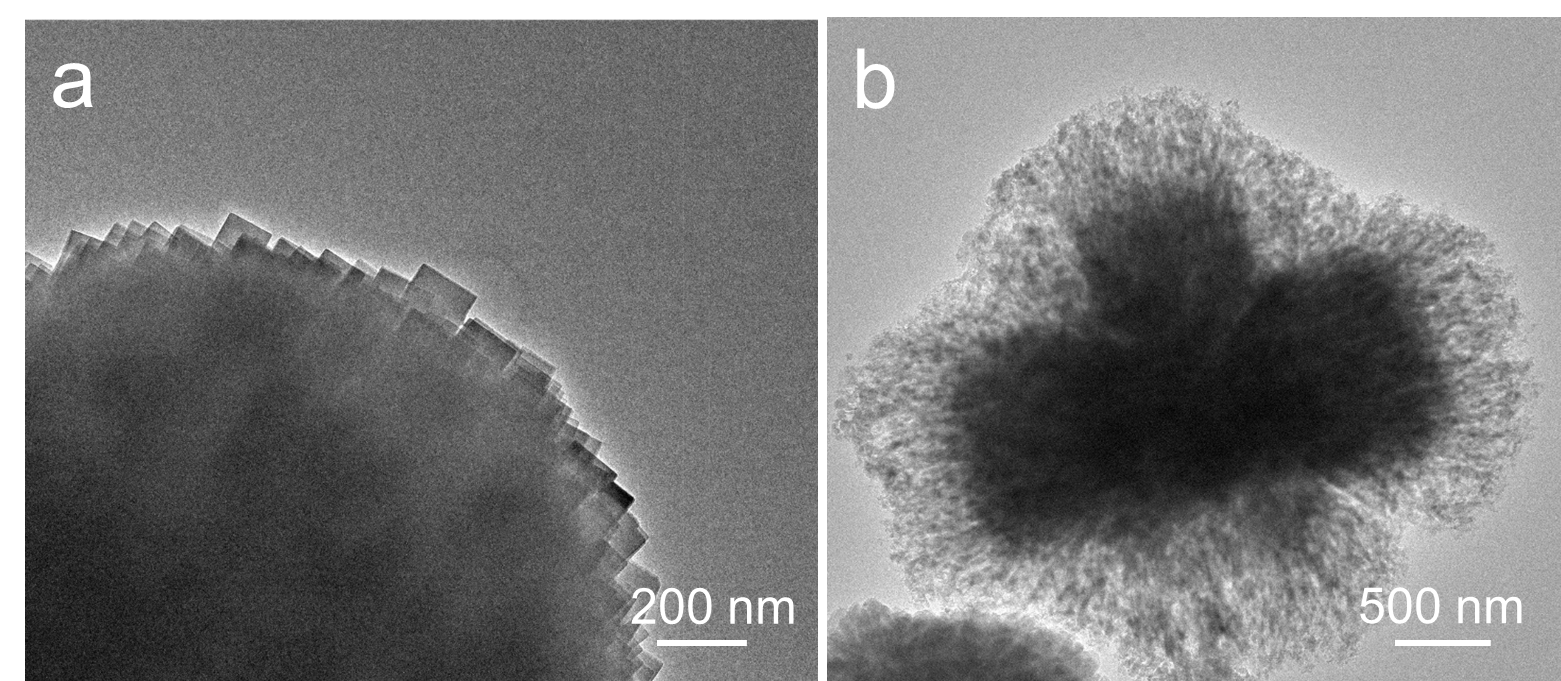


**Figure S1** the TEM images of the parent (Z_P_, a) and NH_4_F treated zeolite crystals (Z_F_, b).

**
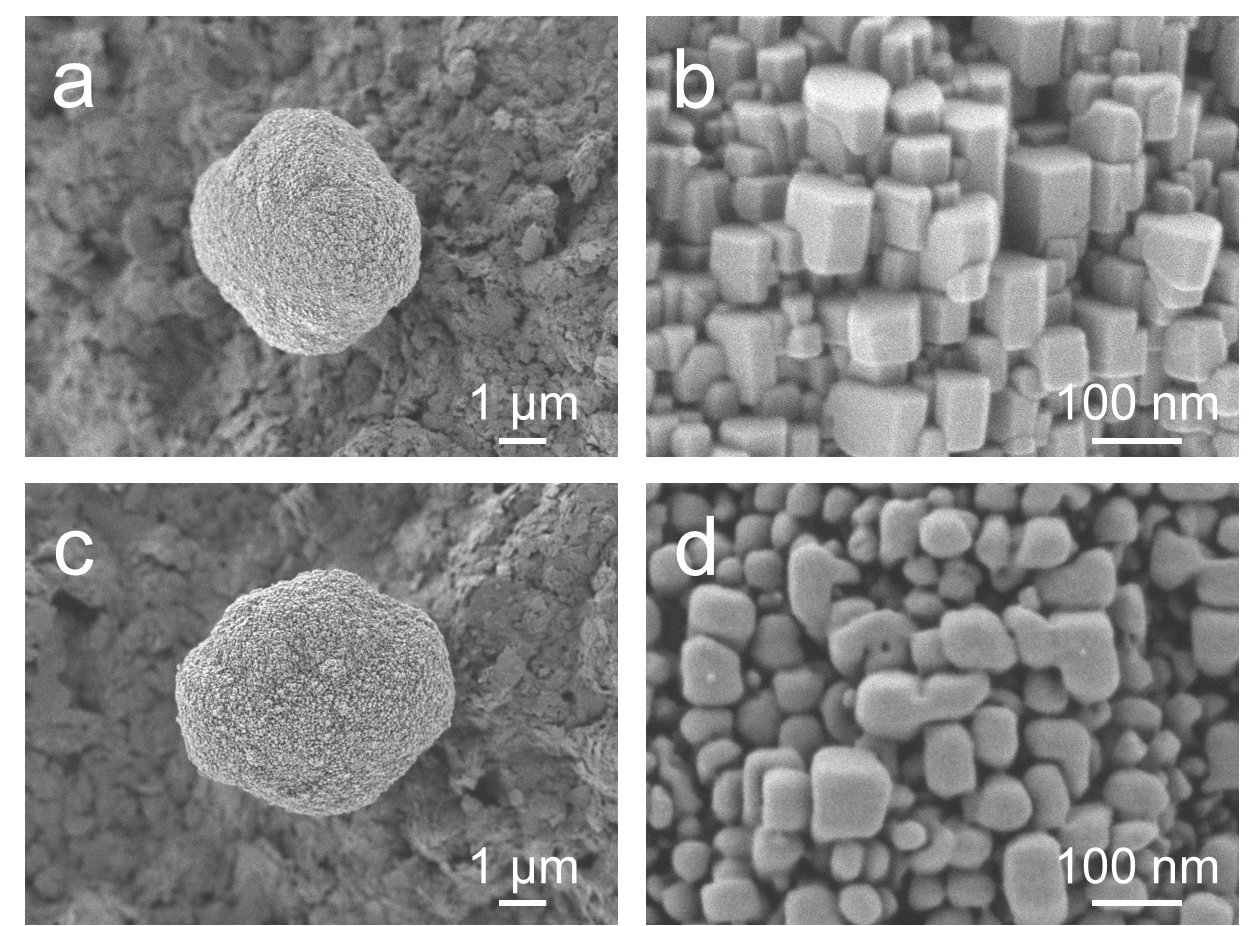
**

**Figure S2** the SEM images of the parent (Z_P_) and NH_4_F treated zeolite crystals (Z_F_). The SEM images were quoted from the work reported by Asahina et al^1^.


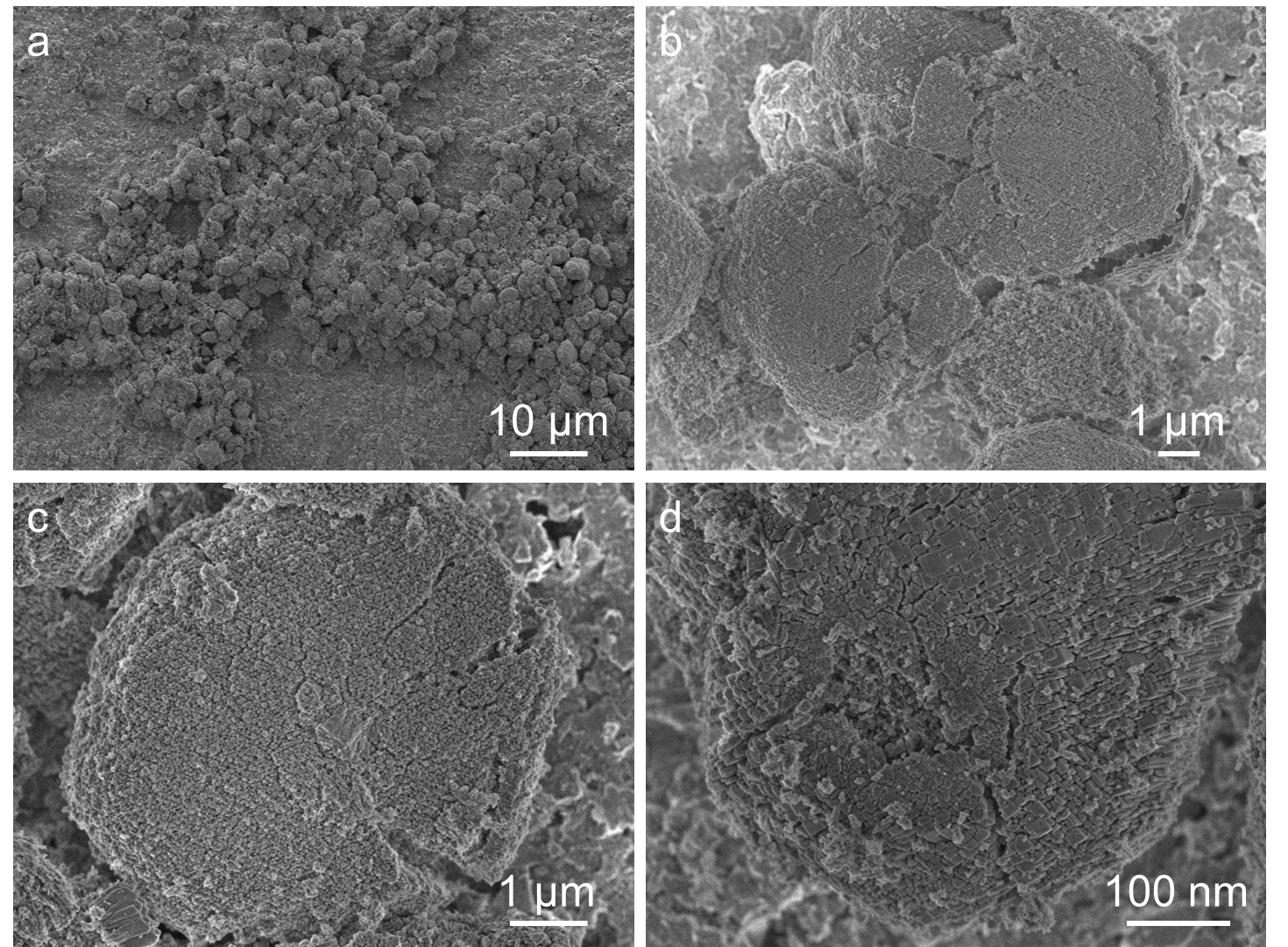


**Figure S3** the SEM images of the NH_4_F treated zeolite crystals (Z_F_).


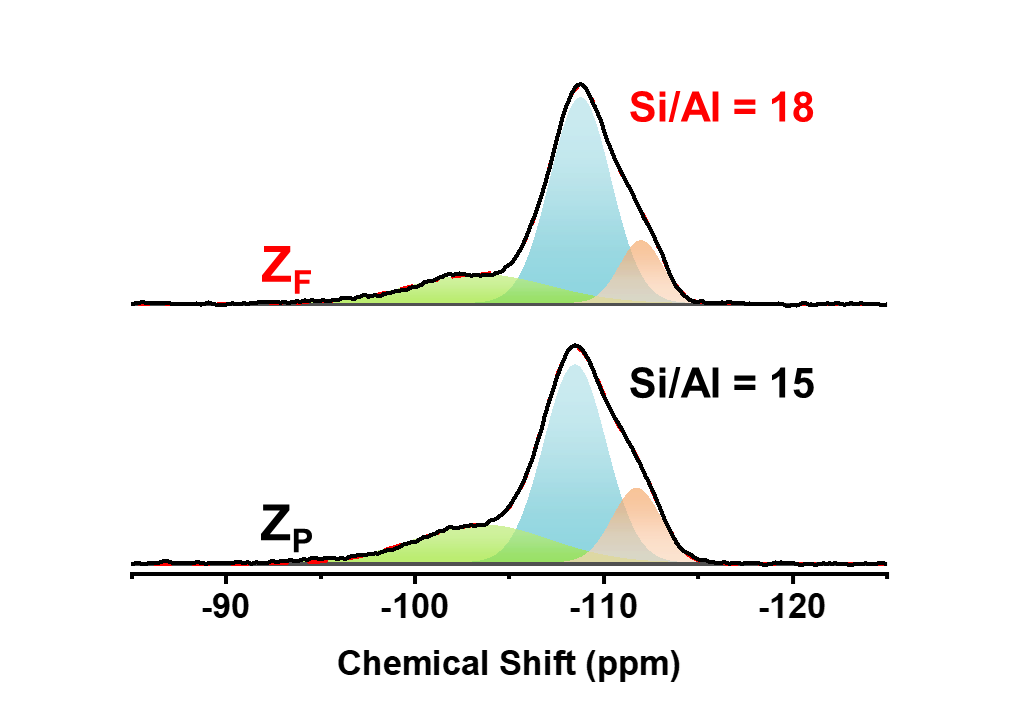


**Figure S4** the SEM images of the NH_4_F treated zeolite crystals (Z_F_).


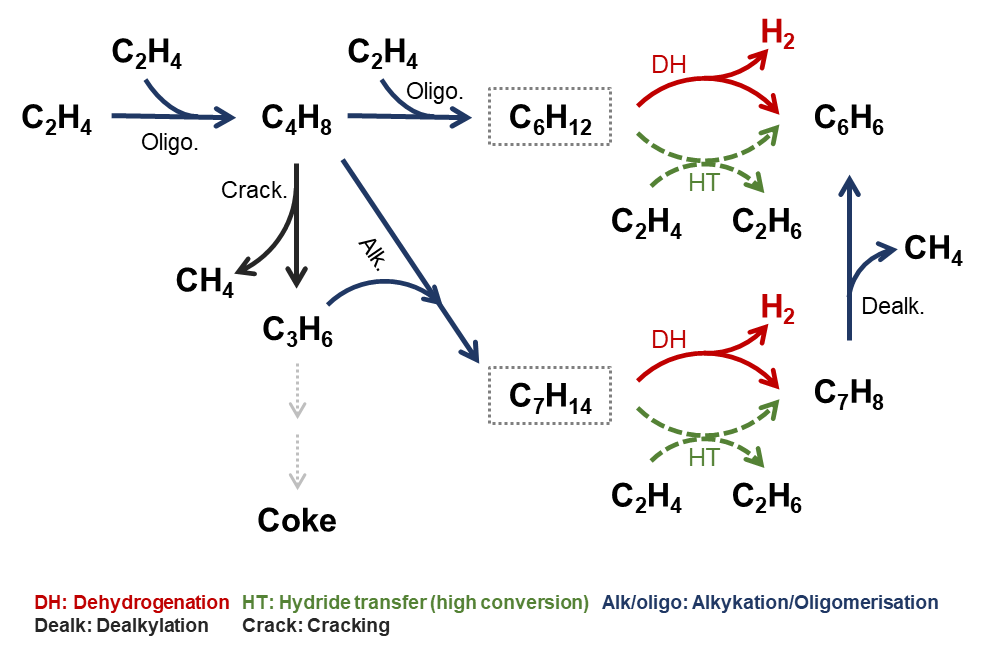


**Figure S5** Proposed reaction scheme of ethylene transformation on H-ZSM-5 zeolite at 700 °C^6^.


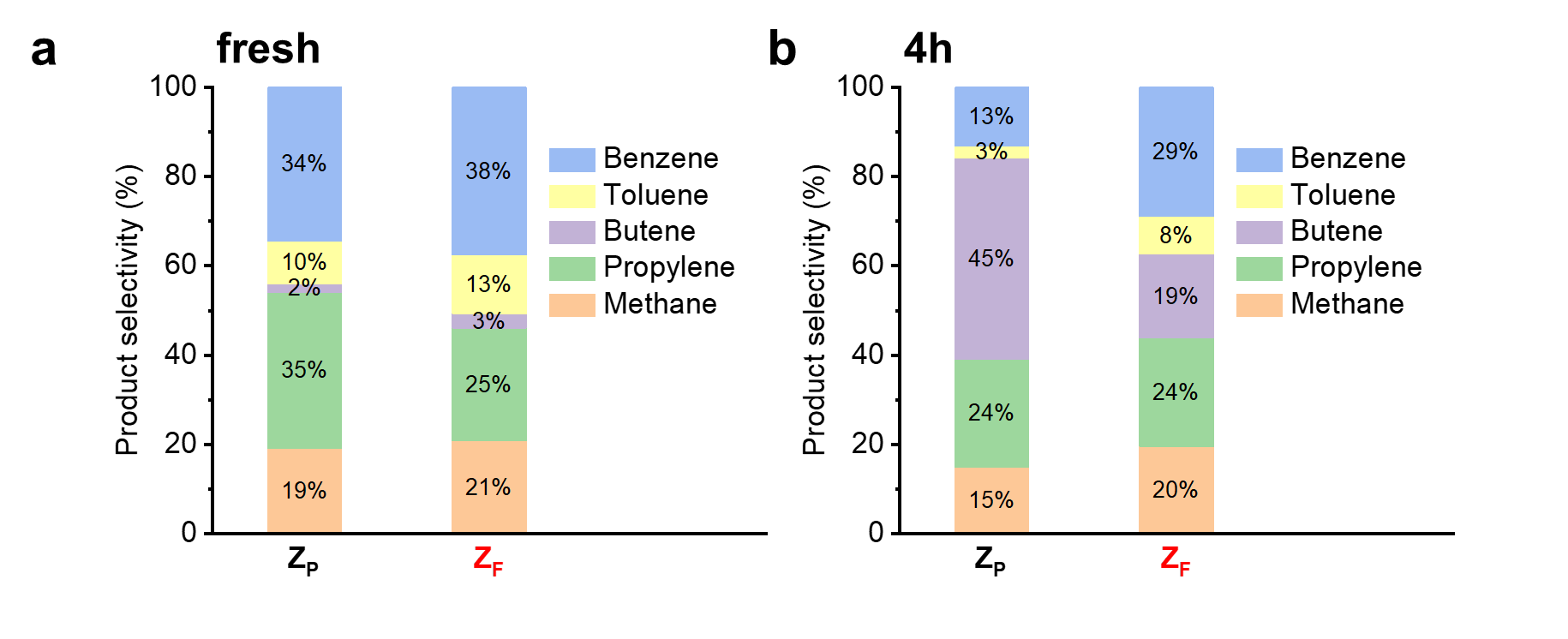


**Figure S6** The product selectivity of ethylene transformation over the parent (Z_P_) and NH_4_F-treated (Z_F_) zeolite catalysts (reaction time: 0 h (a) and 4 h (b)).


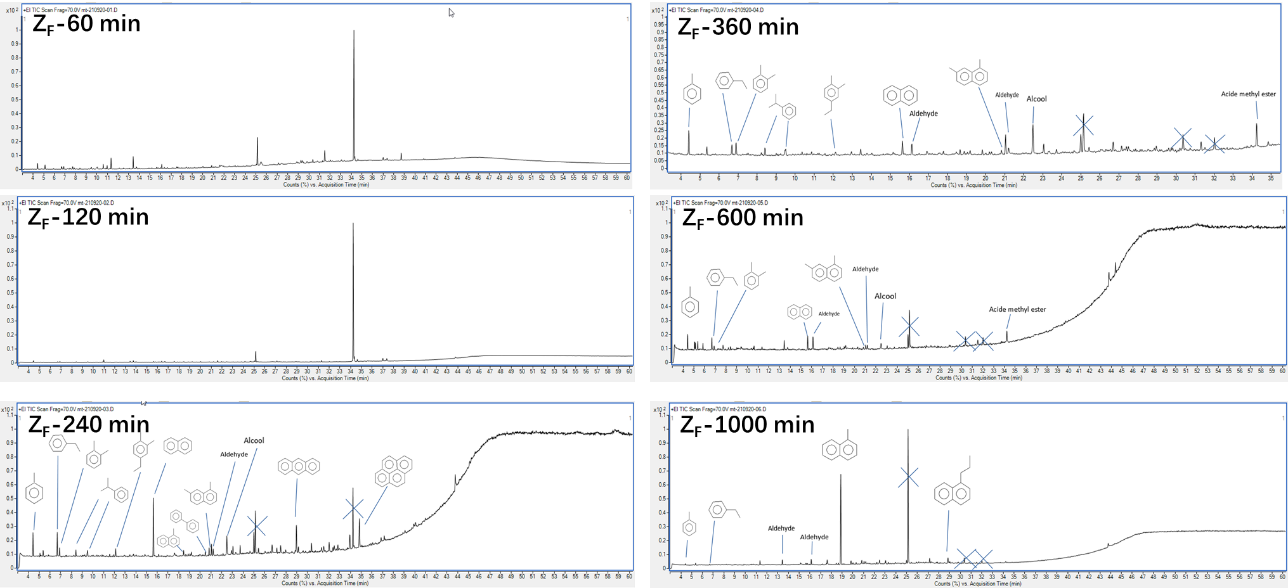


**Figure S7** Evolution of coke species composition on Z_F_ during EDA reaction.


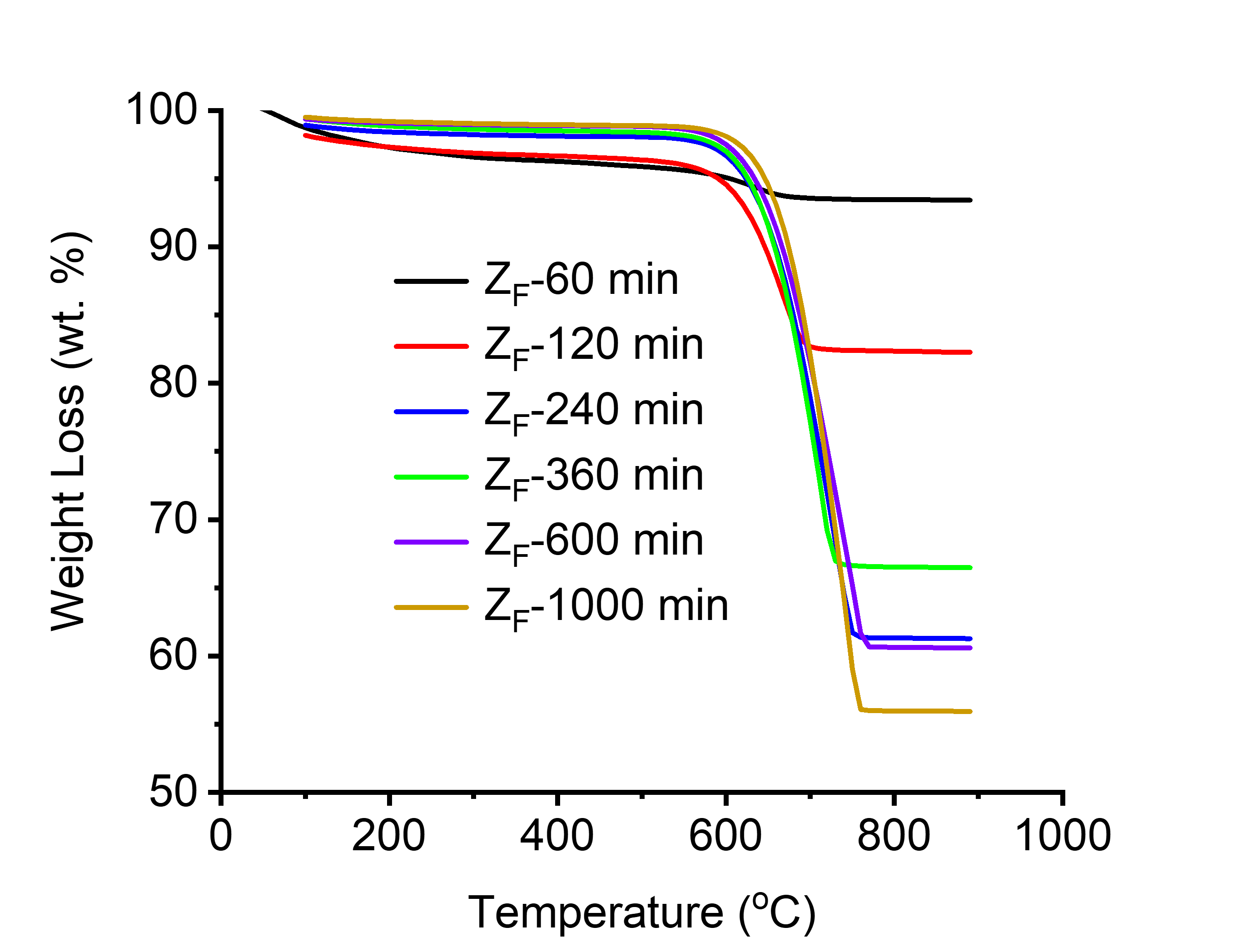


**Figure S8** the TG curves of the Z_F_ with different reaction time.


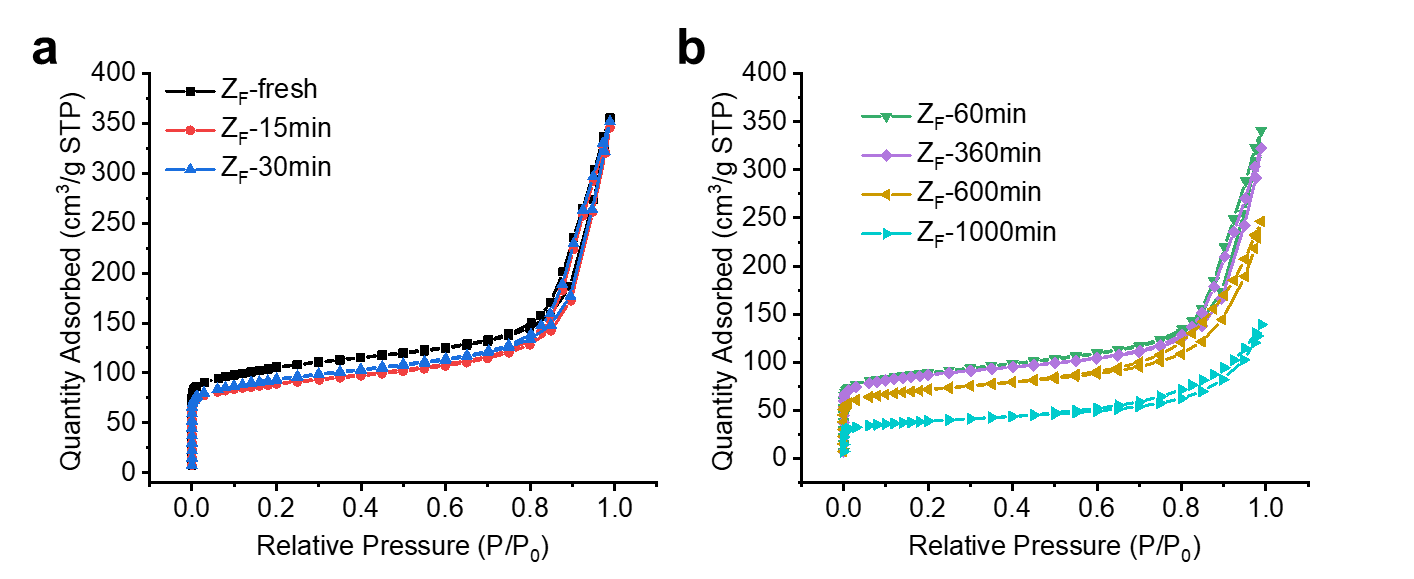


**Figure S9** N_2_ isotherms of the Z_F_ catalysts with various time on stream during EDA reaction.


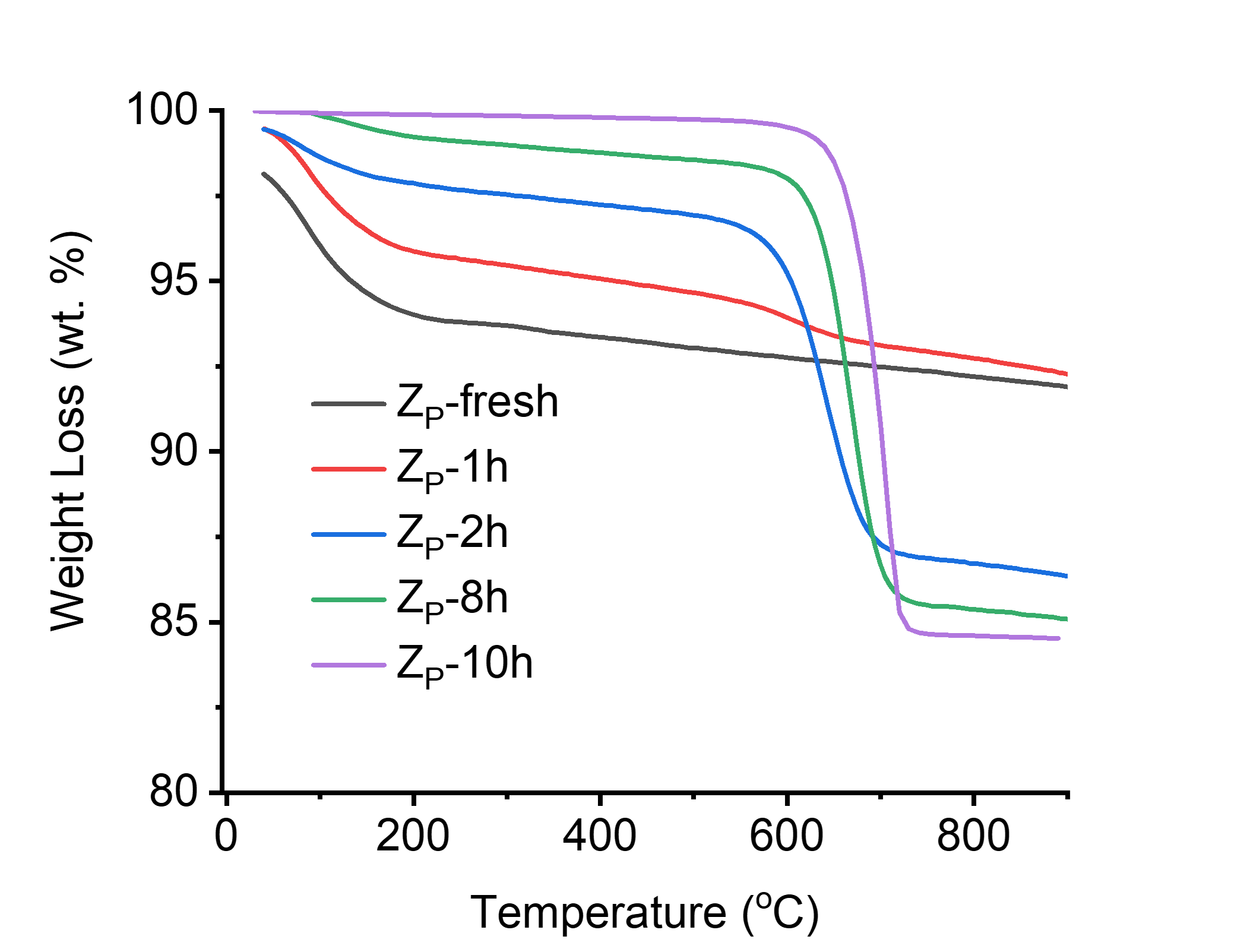


**Figure S10** the TG curve of the Z_P_ with different reaction time.

1. N. Asano, S. Asahina, J. Lu, J. Xu, Y. Shen, Z. Qin, S. Mintova, “Advanced scanning electron microscopy techniques for structural characterization of zeolites” Inorganic Chemistry Frontiers, 2022, 9(16), 4225-4231.

2. S. V. Konnov, F. Dubray, E. B. Clatworthy, C. Kouvatas, J. P. Gilson, J. P. Dath, D. Minoux, C. Aquino, V. Valtchev, S. Moldovan, S. Koneti, N. Nesterenko, S. Mintova, “Novel strategy for the synthesis of ultra‐stable single‐site Mo‐ZSM‐5 zeolite nanocrystals” Angewandte Chemie International Edition, 2020, 132(44), 19721-19728.

3. F. Dubray, S. Moldovan, C. Kouvatas, J. Grand, C. Aquino, N. Barrier, J.-P. Gilson, N. Nesterenko, D. Minoux, S. Mintova, “Direct evidence for single molybdenum atoms incorporated in the framework of MFI zeolite nanocrystals” Journal of the American Chemical Society, 2019, 141(22), 8689-8693.

4. Y. Shen, S. Zhang, Z. Qin, A. Beuque, L. Pinard, S. Asahina, N. Asano, R. Zhang, J. Zhao, Y. Fan, X. Liu, Z. Yan, S. Mintova, “Effect of Mixing Order of Si and Al Sources on the Inner Architecture and Catalytic Performance of ZSM-5 Zeolites” ACS Catalysis, 2024, 14(6), 3766-3777.

5. P. Magnoux, P. Roger, C. Canaff, V. Fouche, N.S. Gnep, M. Guisnet, “New technique for the characterization of carbonaceous compounds responsible for zeolite deactivation” Elsevier, 1987, 34, 317-330.

6. A. Beuque, M. Barreau, E. Berrier, J. Paul, N. Batalha, A. Sachse, L. Pinard, “Transformation of dilute ethylene at high temperature on micro-and nano-sized H-ZSM-5 zeolites” Catalysts, 2021, 11(2), 282.
